# Parliamentary Report on the Dentists Act

**Published:** 1919-03-15

**Authors:** 


### The Hospital

The Workers' Newspaper of Administrative Medicine and Institutional Life, Administration, National Insurance and Health.

No. 1710, Vol. LXV. SATURDAY, MARCH 15, 1919. PRICE TWOPENCE

### PARLIAMENTARY REPORT ON THE DENTISTS ACT.*

Perhaps the gravity of the subject of dental disease is not as apparent as it should be, even to readers of this, a technical medical journal. Therefore, before commenting on the report of the Departmental Committee, which was set up "to inquire into the extent and gravity of dental practice by persons not qualified under the Dentists Act," let us see why it is of such serious importance.

The writer of a letter to the *Times*, commenting on the report, and a leader on the report which had appeared in a previous issue of that paper, expressed it as his opinion "that in the last twenty-five years dental caries and its sequelæ had in the aggregate caused more misery and disease than had either cancer, tubercle, or war, at least amongst our own people." This is, indeed, a very serious statement, and it is probably not an exaggerated one. All classes of all ages and of both sexes are being continually attacked by this, by far the most common of pathological conditions. Well-established caries invites and fosters the establishment of whole colonies of organisms. These in time produce conditions which vary in degree of danger according to the predominance of any one or more of the different varieties of organism present. In pyorrhœa, streptococci, predominating, prepare the way for staphylococci, these being in smaller proportion, the pneumoccus being generally present. Secondary infection may take place of any part of the digestive tract by direct continuity of surface tissue, and the neighbouring structures of nose, tonsils; ears, and throat may be affected. Caries which brings about the death of a tooth, or treatment of caries which involves killing a tooth, lead to another series of troubles. The products of the decomposition of the "dead" tooth's root or roots are apt to get into and soil the blood stream. The soiling of the blood stream may be made manifest in various directions—in the joints most commonly, but also in the nervous system, in the skin, including the hair, the eyes, etc. It has often lead to death itself. Between the above definite trouble and the condition we call health are all the gradations of ill-health which constitute the different stages towards a nameable lesion.

Bearing in mind the foregoing, we can now set out most sympathetically—for we are all in the same condemnation, no one of us escapes dental caries—to examine what is being done by the State for our protection. As a preliminary, let us see what brought about the Dental Act of forty years ago. In a lesser degree, the same chaos existed as exists to-day. Any one could practise so-called dentistry. The Act had as its intention the provision of qualified practitioners, who by curriculum and examination had to satisfy the State that they were efficiently trained. It was quite an expensive education, both as regards money and as regards time. The necessary qualifications in either the Church, Navy, Army, law, or in medicine would have cost no more in either direction. Now, taking the Parliamentary Report itself, we see why, as a profession, dentistry was doomed to be unpopular from the beginning. The legal phraseology used in framing the Act—the fence which was to keep out the unqualified—failed in its object: the fence does not exist! Therefore the social standing accorded to the members of the profession was quite inadequate, and did not repay the expenditure of time and money necessary to the acquisition of its qualification. Whilst the number of the unqualified has increased by leaps and bounds, the number of students seeking qualification under the Act has remained practically stationary.

The Report definitely finds "that there are grave evils associated with the practice of dentistry and dental surgery by persons not qualified under the Dentists Act," and that these have caused a lowering of the social status and public esteem of the dental profession, thereby causing a shortage of registered dentists, grave personal injury to patients, septic mouths, and sacrifice of sound teeth. The unregistered practitioner is held responsible for the existence in the public mind of the idea that there is no advantage in preserving the natural teeth, and that the correct thing is to let these decay, and, when trouble arises, to have the teeth out and have false ones. The registered practitioner is said to be mainly practising amongst the upper and middle classes, and the unregistered practitioner amongst the artizan and working classes. Various remedies are proposed for the dearth of qualified dentists; amongst the many set forth is one by which a process of selection from amongst the present unregistered practitioners is to be adopted. Surely this will be very difficult in view of what the report says of the unregistered practitioners? Elaboration with regard to qualification and title are foreshadowed for the future, so as to allow the public to distinguish between the qualified and registered of to-day and the unqualified but registered of to-morrow. The masses will be absolutely unaffected by these differences in title. It is difficult to imagine that the dental profession as a whole will be helped much should the recommendation of the Committee be adopted. Dental disease remains the most serious question of national health, and the biggest of the problems to be overcome in trying to make a C3 into an A1 people. A new Act, on the lines of the report, will do little towards solving the problem. What is needed is a large addition of trained practitioners, not the influx of numbers of the untrained; and if it be true that the dental profession—as it will be in its proposed amended form—cannot hope to cope with the seriousness of the problem before it, which is the *treatment* of dental caries, would it not be better to have a Royal Commission to inquire into the *causes* of dental caries? It must be acknowledged, however, that the Committee had to investigate and report on a most difficult question.

